# Broad Neutralization of SARS-CoV-2 Variants, Including Omicron, following Breakthrough Infection with Delta in COVID-19-Vaccinated Individuals

**DOI:** 10.1128/mbio.03798-21

**Published:** 2022-03-17

**Authors:** Thomas Lechmere, Luke B. Snell, Carl Graham, Jeffrey Seow, Zayed A. Shalim, Themoula Charalampous, Adela Alcolea-Medina, Rahul Batra, Gaia Nebbia, Jonathan D. Edgeworth, Michael H. Malim, Katie J. Doores

**Affiliations:** a Department of Infectious Diseases, School of Immunology & Microbial Sciences, King’s College London, London, United Kingdom; b Centre for Clinical Infection and Diagnostics Research, Department of Infectious Diseases, Guy’s and St Thomas’ NHS Foundation Trust, London, United Kingdom; The Peter Doherty Institute for Infection and Immunity

**Keywords:** COVID-19, neutralizing antibody, Omicron, SARS-CoV-2, immunization

## Abstract

Numerous studies have shown that a prior SARS-CoV-2 infection can greatly enhance the antibody response to COVID-19 vaccination, with this so called “hybrid immunity” leading to greater neutralization breadth against SARS-CoV-2 variants of concern. However, little is known about how breakthrough infection (BTI) in COVID-19-vaccinated individuals will impact the magnitude and breadth of the neutralizing antibody response. Here, we compared neutralizing antibody responses between unvaccinated and COVID-19-double-vaccinated individuals (including both AZD1222 and BNT162b2 vaccinees) who have been infected with the Delta (B.1.617.2) variant. Rapid production of spike-reactive IgG was observed in the vaccinated group, providing evidence of effective vaccine priming. Overall, potent cross-neutralizing activity against current SARS-CoV-2 variants of concern was observed in the BTI group compared to the infection group, including neutralization of the Omicron (B.1.1.529) variant. This study provides important insights into population immunity where transmission levels remain high and in the context of new or emerging variants of concern.

## INTRODUCTION

COVID-19 vaccines have proven to be critical in controlling SARS-CoV-2 infections worldwide. Vaccines based on the SARS-CoV-2 Wuhan-1 spike protein generate neutralizing antibodies which constitute an important component of the protective capacity of COVID-19 vaccines. Since the beginning of the global pandemic, variants of SARS-CoV-2 have arisen which encode mutations in the spike protein. Until November 2021, the dominant circulating variant was B.1.617.2 (Delta), but B.1.1.529 (Omicron) is rapidly increasing globally (https://www.who.int/docs/default-source/coronaviruse/2022-01-07-global-technical-brief-and-priority-action-on-omicron---corr2.pdf?sfvrsn=918b09d_20). There is concern that SARS-CoV-2 variants of concern (VOCs) might lead to a reduction in vaccine efficacy, in particular against Omicron, which encodes 31 amino acid changes in the spike protein.

To generate high titers of spike-reactive IgG with potent neutralization, double vaccination is required for both the BNT162b2 (based on mRNA encoding a stabilized spike) and AZD1222 (based on a chimp adenovirus-encoded spike) vaccines (1, 2). Importantly, several studies have shown that SARS-CoV-2 infection prior to vaccination can boost antibody titers and neutralizing activity, with this so-called hybrid immunity leading to greater neutralization breadth against SARS-CoV-2 VOCs (3–7). However, little is known about how breakthrough infection (BTI) in COVID-19-double-vaccinated individuals will impact the magnitude and breadth of the neutralizing antibody response (8–10), particularly in the face of the Omicron variant, where preliminary data show that a 3rd vaccine dose is required for robust neutralization activity (11–15) and predicted for high vaccine efficacy (16). This information would provide important insights into population immunity in areas where transmission levels remain high and where Omicron is rapidly becoming the dominant strain. Here, we compared the magnitude and breadth of the antibody response in individuals infected with the SARS-CoV-2 Delta VOC (vaccine-naive) to the antibody response in individuals who were double vaccinated prior to Delta infection (breakthrough infection [BTI]).

## RESULTS

### Cohort description.

We identified 42 individuals admitted to St Thomas’ hospital who had previously received two COVID-19 vaccinations and subsequently tested positive for COVID-19 (BTI group). We note that at the time of writing, from the patients admitted to St Thomas’ Hospital with COVID-19 since the emergence of Delta (*n* = 635), 260 cases out of 332 (78%) where vaccination was known were either unvaccinated or partially vaccinated (one inoculation). In this study, 30/42 (71%) of patients in the BTI group were admitted to hospital due to COVID-19, of which 11/30 (37%) patients experienced severe disease (severity 4 to 5), and 29/30 (97%) patients had underlying health conditions that predispose to severe disease and were aged between 20 and 103 years (median age, 77 years; interquartile range [IQR], 59 to 86) (Table S1). The remaining 12 participants in the BTI group (29%) were asymptomatic and admitted for reasons other than COVID-19. Asymptomatic patients were aged between 24 and 96 years (median age, 62 years; IQR, 37 to 72). Overall, the BTI group included individuals receiving both the AZD1222 vaccine (*n* = 23) and the BNT162b2 vaccine (*n* = 19). Discarded serum samples were collected between 0 and 53 days post-onset of symptoms (POS), and longitudinal serum samples were collected where possible. The number of days post-second vaccine ranged from 29 to 179 days (median, 109 days).

10.1128/mbio.03798-21.3 TABLE S1 Demographics for the BTI group. Download Table S1, PDF file, 0.1 MB. Copyright © 2022 Lechmere et al. 2022 Lechmere et al. https://creativecommons.org/licenses/by/4.0/ This content is distributed under the terms of the Creative Commons Attribution 4.0 International license.

Sera (*n* = 19) were also collected from vaccine naive individuals admitted to St Thomas’ Hospital due to COVID-19 who had a confirmed infection with the SARS-CoV-2 Delta variant and experienced a range of disease severities, with 9/19 (47%) patients experiencing severe disease (severity 4 to 5). The unvaccinated group was younger than the BTI group (aged between 25 and 82 years [median age, 39 years; IQR, 30 to 51]) and unlike the BTI group, only 9/19 (47%) had underlying health conditions (Table S2). Sera were collected between 12 and 22 days POS.

10.1128/mbio.03798-21.4 TABLE S2 Demographics for the vaccine naive group. Download Table S2, PDF file, 0.07 MB. Copyright © 2022 Lechmere et al. 2022 Lechmere et al. https://creativecommons.org/licenses/by/4.0/ This content is distributed under the terms of the Creative Commons Attribution 4.0 International license.

### IgG and IgM to spike in breakthrough infection.

**FIG 1:**
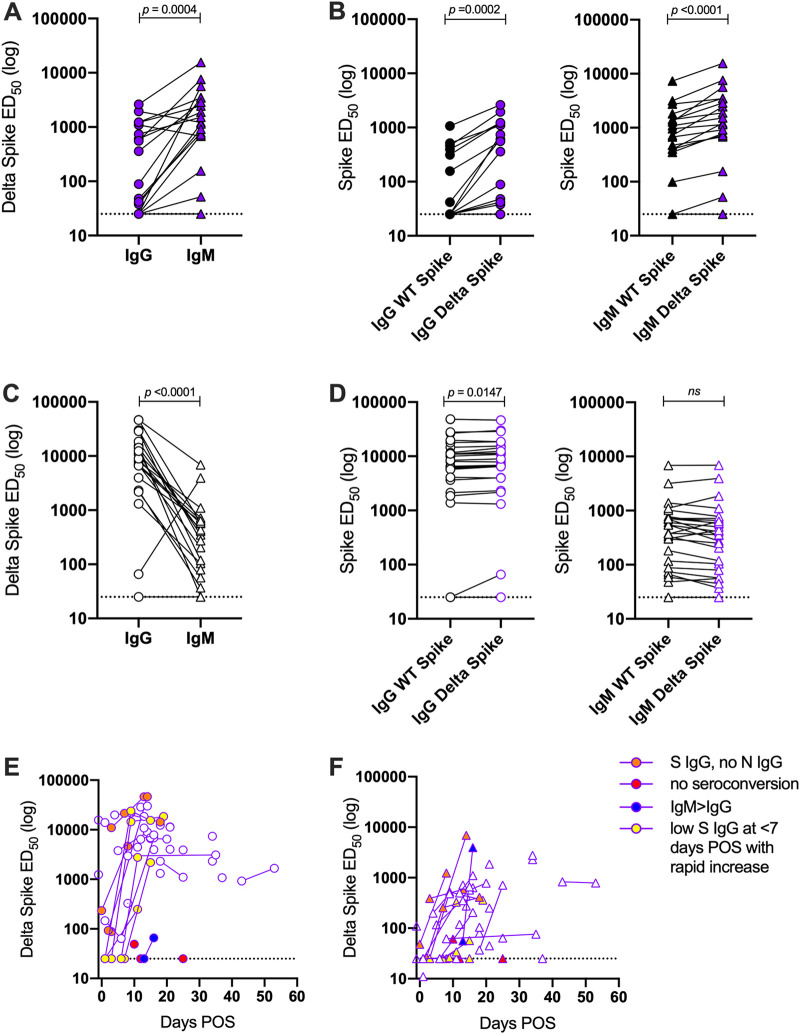
Differences in antibody binding between Delta-infected individuals and COVID-19-vaccinated individuals experiencing delta breakthrough infection. (A) Difference in IgG and IgM titers for sera collected 12 to 22 days POS for the Delta infection (vaccine-naive) group. (B) Comparison of the IgG and IgM ED_50_ values against recombinant WT and Delta spikes for the vaccine-naive group. (C) Difference in IgG and IgM titers for sera for the BTI group. Sera were collected 12 to 22 days POS. (D) Comparison of the IgG and IgM ED_50_ values against recombinant WT and Delta spikes for the BTI group. (E) Longitudinal IgG ED_50_ against recombinant Delta spike in the BTI group. (F) Longitudinal IgM ED_50_ against recombinant Delta spike in the BTI group. Donors with IgM > IgG are shown in blue, donors who did not seroconvert are shown in red, donors with high spike IgG but no N IgG at <7days POS are shown in orange, and donors with low spike IgG at <7 days POS that rapidly increased are shown in yellow. *P* values were calculated using a Wilcoxon matched-pair signed rank test.

First, we measured the IgG and IgM responses to recombinant spike (both wild-type [WT] and Delta) in the two groups by enzyme-linked immunsorbent assay (ELISA). Initial analysis was conducted on sera collected 2 to 3 weeks POS, as we had previously observed peak antibody levels in this time window (17). Sera from unvaccinated individuals infected with the Delta variant at 12 to 22 days POS had higher Delta spike IgM levels than Delta spike IgG (Fig. 1A), indicative of a primary immune response. Statistically higher IgG and IgM titers were observed against the Delta recombinant spike compared to WT spike (Fig. 1B).

For sera collected 12 to 22 days POS in the BTI group, Delta spike IgM levels in the BTI group were lower than the Delta spike IgG level (Fig. 1C), indicative of a recall response. A similar trend was observed for both AZD1222- and BNT162b2-vaccinated individuals (Fig. S1A). Where sequential serum samples were collected, nine individuals had an undetectable or a very low spike IgG response at the earliest time point POS (Fig. 1D and Fig. S1B). However, high titers of spike-specific IgG were detected several days later with only modest increases in IgM titers (Fig. 1E and F). Six donors had IgG against spike at early time points but lacked IgG to the SARS-CoV-2 nucleoprotein (Fig. S1C and D). Although this may provide insight into spike IgG levels prior to infection, it is more likely due to a rapid spike IgG recall response compared to a *de novo* IgG response to nucleoprotein (Fig. 1E and F). One participant (a renal transplant patient) had a high IgM response and low IgG response, similar to the vaccine-naive group, which suggests failed vaccine priming (Fig. 1C). Interestingly, unlike the vaccine-naive group, the IgG and IgM titers against the WT and Delta spikes were comparable in the BTI group (Fig. 1D).

10.1128/mbio.03798-21.1 FIG S1 (A) Comparison of the IgG and IgM ED_50_ values against recombinant WT and Delta spikes for the AZD1222- and BNT162b2-vaccinated individuals experiencing breakthrough infection (BTI). *P* values were calculated using a Wilcoxon matched-pair signed rank test. (B) Longitudinal IgG and IgM ED_50_ against recombinant WT spike in the BTI group. IgG is shown with a circle, and IgM is shown with a triangle. Donors with IgM > IgG are shown in blue, donors who did not seroconvert are shown in red, donors with high Spike IgG but no N IgG at <7 days POS are shown in orange, and donors with low Spike IgG at <7 days POS that rapidly increased are shown in yellow. (C) Longitudinal IgG response to N protein. The dotted line represents the cutoff used to determine N seropositivity. (D) Correlation between ED_50_ against recombinant WT spike in the BTI group and the optical density (OD) for N IgG binding. The horizontal dotted line represents the lowest dilution used in the neutralization assay. The vertical dotted line represents the cutoff used to determine N seropositivity. Data points are color-coded based on the days POS as indicated in the key. The red box highlights samples that have high IgG binding ED_50_ but low IgG to N. Download FIG S1, TIF file, 1.3 MB. Copyright © 2022 Lechmere et al. 2022 Lechmere et al. https://creativecommons.org/licenses/by/4.0/ This content is distributed under the terms of the Creative Commons Attribution 4.0 International license.

Overall, these results indicate a rapid recall response due to prior vaccination in the BTI group and a primary immune response in the vaccine-naive group.

### Neutralization activity following breakthrough infection.

**FIG 2:**
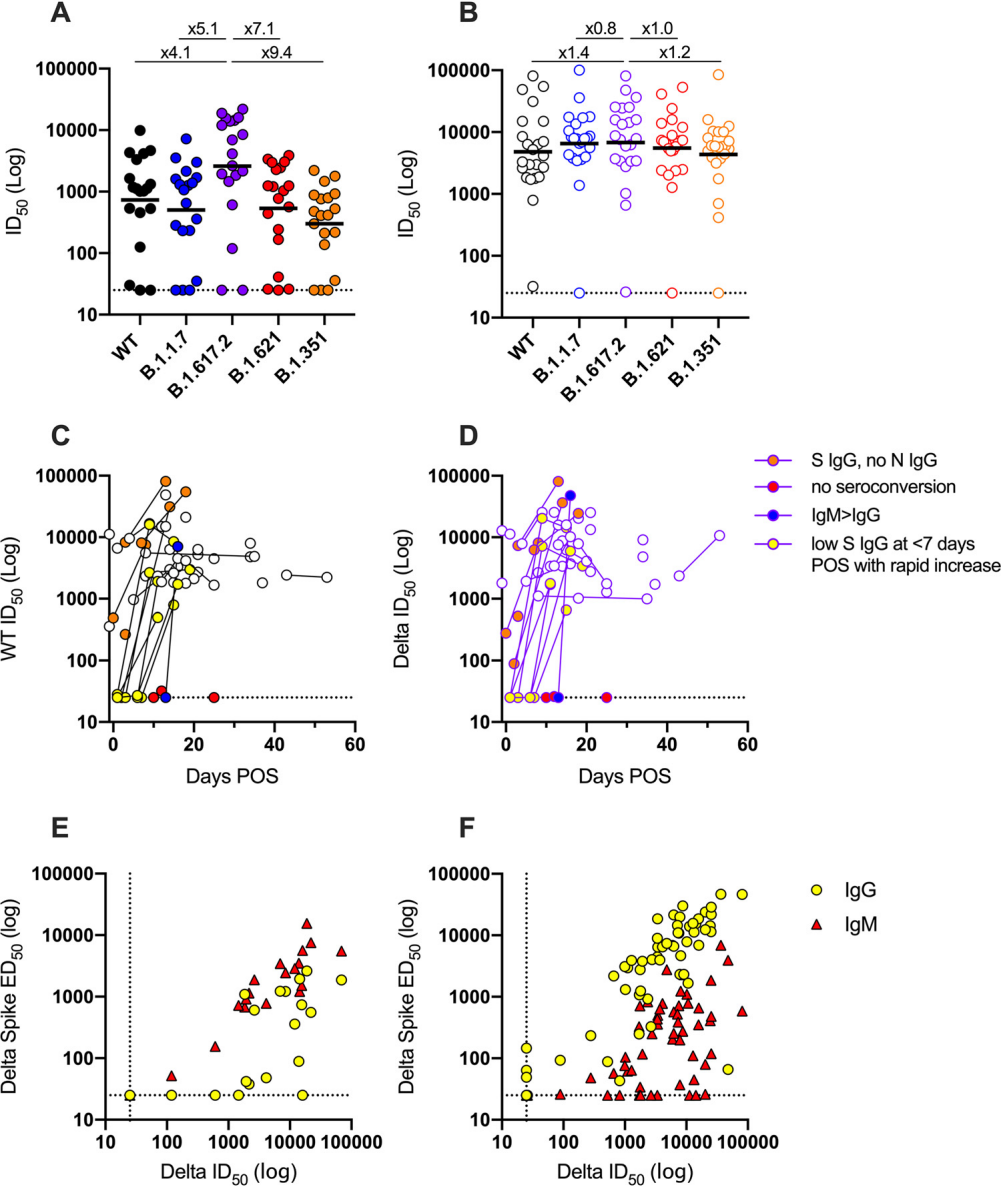
Differences in neutralizing antibody response between Delta-infected individuals and COVID-19-vaccinated individuals experiencing Delta breakthrough infection. (A and B) ID_50_ of neutralization against WT (black) and VOCs Alpha (B.1.1.7, blue), Delta (B.1.617.2, purple), Mu (B.1.621, red), and Beta (B.1.351, orange) for sera from (A) SARS-CoV-2 vaccine-naive, Delta-infected individuals and (B) BTI individuals. Samples were collected 12 to 22 days POS. Black lines show the GMT. Fold decreases in GMT compared to Delta are shown above. Neutralization assays were carried out in duplicate. (C and D) Longitudinal neutralization potency of sera from BTI individuals against (C) WT pseudovirus particles and (D) Delta pseudovirus particles. Donors with IgM > IgG are shown in blue, donors who did not seroconvert are shown in red, donors with high Spike IgG but no N IgG at <7 days POS are shown in orange, and donors with low Spike IgG at <7 days POS that rapidly increased are shown in yellow. Data for the Alpha, Beta, and Mu VOCs are shown in Fig. S2A. (E and F) Correlation (Spearman, *r*) between ID_50_ of neutralization and IgM or IgG ED_50_ for Delta spike binding for (E) Delta-infected individuals (IgM: *r* = 0.92, *r*^2^ = 0.90, *P* < 0.0001; IgG: *r* = 0.66, r^2^ = 0.43, *P* = 0.001) and (F) COVID-19-vaccinated individuals experiencing breakthrough infection (IgM: *r* = 0.61, r^2^ = 0.38, *P* < 0.0001; IgG: *r* = 0.83, r^2^ = 0.75, *P* < 0.0001). A linear regression was used to calculate the goodness of fit (r^2^). The dotted lines represent the lowest serum dilution used in each assay. IgG is shown with yellow circles, and IgM is shown with red triangles.

Next, we measured neutralization breadth and potency in the two groups using HIV-1 (human immunodeficiency virus type 1)-based virus particles, pseudotyped with SARS-CoV-2 spike from different VOCs (wild-type [Wuhan], alpha [B.1.1.7], Delta [B.1.617.2], mu [B.1.621], and beta [B.1.351]) and a HeLa cell line stably expressing the ACE2 receptor (17). The majority (17/19, 89%) of the vaccine-naive group produced a robust homologous neutralizing response against the Delta VOC (Fig. 2A). Cross-neutralization of the parental strain and other VOCs was detected for most individuals, albeit at a reduced potency. As we have reported previously (18), the greatest reduction was observed against beta with a 9.4-fold reduction in the geometric mean titer (GMT), reflecting greater antigenic distance.

10.1128/mbio.03798-21.2 FIG S2 **(**A) Neutralization potency of sera following breakthrough infection at different days post-onset of symptoms (POS) against Alpha, Beta, and Mu pseudovirus particles. (B) ID_50_ of neutralization against WT (black) and VOCs Alpha (blue), Delta (purple), Mu (red), and Beta (orange) for sera collected 12 to 22 days POS from AZD1222-vaccinated (*n* = 16) or BNT162b2 (*n* = 10) individuals experiencing SARS-CoV-2 Delta breakthrough infection. The black line shows the geometric mean titer. Download FIG S2, TIF file, 1.1 MB. Copyright © 2022 Lechmere et al. 2022 Lechmere et al. https://creativecommons.org/licenses/by/4.0/ This content is distributed under the terms of the Creative Commons Attribution 4.0 International license.

Sera collected between 12 and 22 days POS from individuals in the BTI group showed a robust homologous neutralizing response as well as strong cross-neutralization of the parental variant and VOCs (Fig. 2B). Only a 1.2-fold reduction in GMT was observed against the more neutralization-resistant beta VOC. Several individuals in the BTI group with sera collected soon after onset of symptoms showed no or very low neutralization against both WT and Delta variants; however, potent neutralizing activity was detected several days later (Fig. 2C and D and Fig. S2A). Geometric mean titers against the five variants were very similar between AZD1222- and BNT162b2-vaccinated individuals (Fig. S2B). Three participants in the BTI group either failed to produce neutralizing antibodies or had titers close to baseline despite vaccination and SARS-CoV-2 infection (Fig. 2C and D). These individuals had underlying health conditions including cancer (one participant was undergoing rituximab treatment) and type-2 diabetes.

As would have been anticipated, IgG half-maximal binding dilution (ED_50_) values correlated best with the serum dilution that inhibits 50% infection (ID_50_) values for the BTI group, whereas IgM ED_50_ values correlated best with ID_50_ values for the vaccine-naive group (Fig. 2E and F), further highlighting the priming capacity of both the AZ1222 and BNT162b2 vaccines.

### BTI generates neutralizing activity against Omicron.

**FIG 3:**
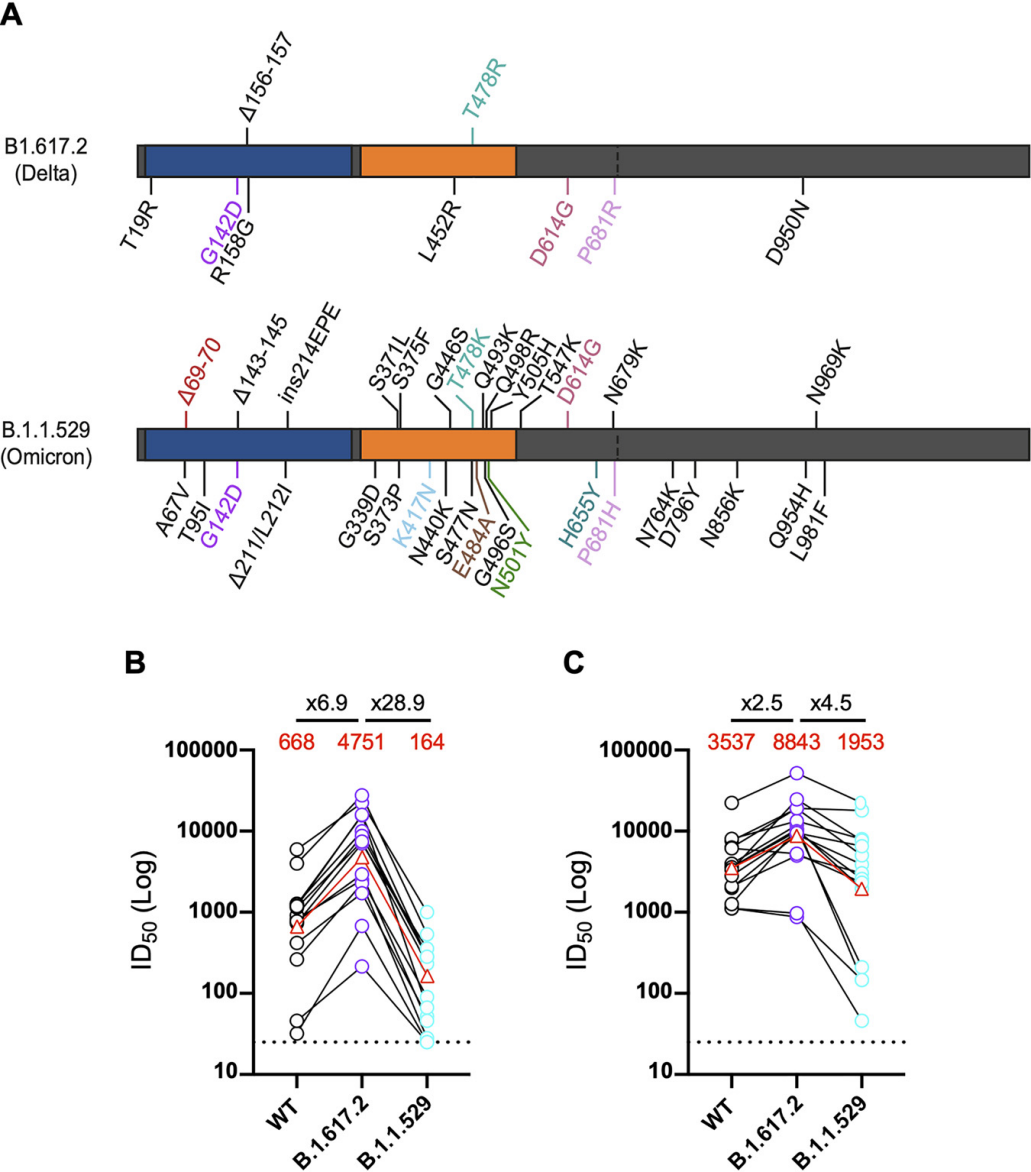
Neutralization of omicron in BTI and delta-infected individuals. (A) Schematic showing mutations in the Delta (B.1.617.2) and Omicron (B.1.1.529) spikes. (B and C) Select sera from (B) SARS-CoV-2 Delta-infected (vaccine-naive) individuals (*n* = 14, 13 to 22 days POS) and (C) BTI individuals (*n* = 15, 12 to 21 days POS) were tested against WT, Delta, and Omicron VOCs. The ID_50_ of neutralization against WT (black) and VOCs Delta (purple), and Omicron (turquoise) for each participant are linked. Geometric mean titers against WT, Delta, and Omicron VOCs are shown in red. The GMT and fold decrease in GMT against omicron compared to WT and delta are shown above. Neutralization assays were carried out in duplicate.

In November 2021, Omicron was identified that encoded 31 amino acid mutations in the spike protein (Fig. 3A). Initial studies suggest that these mutations lead to large reductions in neutralization of sera from double-vaccinated individuals. However, administration of a third vaccine dose greatly enhances neutralization titers against Omicron, suggesting incomplete neutralization escape (11–15, 19). Neutralization activity of a subset of 14 sera from the vaccine-naive group and 15 sera from the BTI group were measured against WT, Delta, and Omicron variants (Fig. 3B). In Delta-infected vaccine-naive individuals, a 28.9-fold reduction in GMT against Omicron compared to Delta was measured compared to a 6.9-fold reduction in GMT for WT. Sera from two participants did not neutralize the Omicron variant at the lowest dilution point (1:25). In contrast, all 15 sera from the BTI group neutralized the Omicron variant with only a 4.5-fold reduction in GMT against Omicron compared to Delta GMT (Fig. 3C). Three BTI individuals showed a 21- to 81-fold reduction in the ID_50_ against Omicron compared to the ID_50_ against Delta, all of which were receiving treatment for underlying health conditions. These results further highlight the breadth of the neutralizing antibody response following BTI with the Delta variant.

## DISCUSSION

These data demonstrate that while 2 doses of COVID-19 vaccine (both BNT162b2 or AZD1222) was not sufficient to provide sterilizing immunity against SARS-CoV-2 infection in these particular individuals, breakthrough infection generated a strong anamnestic response. Although this study cannot provide information on the titer of neutralizing antibody required for protection against infection with the Delta VOC, longitudinal sampling revealed that six participants who had undetectable neutralization or an ID_50_ of ∼25 against the Delta VOC at the earliest time point sampled rapidly developed IgG to spike and serum-neutralizing activity upon infection, showing that both AZD1222 and BNT162b2 vaccination primed their immune system to respond rapidly upon SARS-CoV-2 infection. In the BTI group, 30/42 (71%) were admitted to hospital due to COVID-19, the median age was 77 years, and only 1/30 (3%) had no comorbidities that would predispose to severe disease. This suggests that the individuals admitted with BTI were at particular risk of severe disease due to advancing age and/or comorbidities. Indeed, advancing age was the main criterion on which vaccination schedule was based in the United Kingdom, meaning that those over 70 years were among the first to be offered vaccination in January 2021. As such, vaccine-induced immunity may have waned in this group due to the longer interval between vaccination and exposure, facilitating subsequent BTI (20–23). Indeed, the median time elapsed since last vaccination in the BTI group was 109 days, with 24/30 (80%) being vaccinated over 10 weeks prior to symptom onset. Others have described waning of vaccine-induced immunity against Delta after 10 weeks, especially in older age groups (24, 25). Notably, the vaccine-naive group was much younger, and a large proportion had no comorbidities.

When comparing the antibody response of the BTI group and the vaccine-naive group, we observe that prior vaccination led to a more potent and broader neutralizing antibody response during the acute phase of infection, including against the highly mutated Omicron variant. As we do not have matched sera collected prior to breakthrough infection, we cannot comment on the breadth of the neutralizing antibody response prior to infection. However, in this BTI vaccinated cohort, boosting is occurring with a heterologous spike which may contribute to the broadening of the serum neutralizing activity. In addition, all individuals in this study received an extended booster regime (8 to 12 weeks postprime) which has been suggested to generate a broader response than the short (3 to 4 week) boost regime (26, 27). Further studies examining the antibody response at the monoclonal level are needed to understand the mechanisms underlying the enhanced neutralization breadth. Broader serum activity could arise from vaccine-derived antibodies that gain neutralization breadth upon exposure to Delta spike or a *de novo* response directed against the Delta spike. Broadening of the neutralizing antibody response has been reported at later time points following natural infection (∼6 to 10 months) (18, 28), and therefore, despite narrow serum neutralization breadth in the vaccine-naive group, convalescent-phase sera collected at later time points would be expected to have broader neutralizing activity. The large decrease in neutralization of viral particles pseudotyped with Omicron spike by sera from Delta-infected individuals highlights the large antigenic distance between the Delta and Omicron spike glycoproteins and suggests that unvaccinated individuals infected with Delta VOC may have low protection against infection with the Omicron VOC (18, 29).

Although the Omicron VOC is more neutralization resistant, several studies have reported smaller fold-reductions in serum neutralization potency for Omicron following 3 doses of COVID-19 vaccination (range, 4- to 7-fold) compared to those who had received only 2 vaccine doses (range, 20- to >40-fold) (11–15, 30). Overall, the data presented here suggest that a breakthrough SARS-CoV-2 Delta infection is also acting as an effective booster which could provide broad protection against current VOCs, including Omicron. Therefore, this study provides important insights into population immunity in double-COVID-19-vaccinated individuals where SARS-CoV-2 transmission levels remain high. A limitation of this study is that it does not measure the durability of the neutralizing antibody response following BTI or how BTI affects memory B and T cell responses. Further research is needed to determine how broadening of the neutralizing antibody response translates into protection from reinfection and/or disease. As new VOCs arise with new/unique combinations of mutations, our data suggest that a broad neutralizing antibody response generated by a combination of vaccination and infection may provide immunity against other/emerging VOCs.

## MATERIALS AND METHODS

### Ethics.

Collection of surplus serum samples was approved by South Central-Hampshire B REC (20/SC/0310). SARS-CoV-2 cases were diagnosed by reverse transcriptase PCR (RT-PCR) of respiratory samples at St Thomas’ Hospital, London, UK. Sera were selected based on the availability of longitudinal samples and knowledge of timing and type of COVID-19 vaccination.

### COVID-19 severity classification.

Disease severity was determined as previously described (17, 18). Patients diagnosed with COVID-19 were classified as follows: (0) asymptomatic or no requirement for supplemental oxygen; (1) requirement for supplemental oxygen (fraction of inspired oxygen [*F*iO_2_] < 0.4) for at least 12 h; (2) requirement for supplemental oxygen (*F*iO_2_ ≥ 0.4) for at least 12 h; (3) requirement for noninvasive ventilation/continuous positive airway not a candidate for escalation above level 1 (ward-based) care; (4) Requirement for intubation and mechanical ventilation or supplemental oxygen (*F*iO_2_ > 0.8) and peripheral oxygen saturations <90% (with no history of type 2 respiratory failure [T2RF]) or <85% (with known T2RF) for at least 12 h; (5) requirement for extracorporeal membrane oxygenation (ECMO).

### Virus sequencing.

Delta variant infection was confirmed using whole-genome sequencing as previously described (18) or using multiplex tandem-PCR (MT-PCR) (31).

### Plasmids.

WT, B.1.1.7, B.1.351, B.1.621, B.1.617.2, and B.1.1.529 codon-optimized spike plasmids were obtained from Wendy Barclay (Imperial College London). The final 19 amino acids were removed using a K1255* mutation. The B.1.1.7 mutations introduced were ΔH69/V70, ΔY144, N501Y, A570D, D614G, P681H, T716I, S982A, and D1118H. The B.1.351 mutations introduced were D80A, D215G, Delta242-244, R246I, K417N, E484K, N501Y, D614G, and A701V. The B.1.617.2 mutations introduced were T19R, G142D, Δ156-157, R158G, L452R, T478R, D614G, P681R, and D950N. The B.1.621 mutations introduced were T95I, Y144T/144insS/Y145N, R346K, E484K, N501Y, D614G, P681H, and D950N. The B.1.1.529 mutations introduced were A67V, Δ69-70, T95I, G142D/Δ143-145, Δ211/L212I, ins214EPE, G339D, S371L, S373P, S375F, K417N, N440K, G446S, S477N, T478K, E484A, Q493K, G496S, Q498R, N501Y, Y505H, T547K, D614G, H655Y, N679K, P681H, N764K, D796Y, N856K, Q954H, N969K, and L981F.

### Glycoprotein expression and purification.

The recombinant wild-type (Wuhan-1 strain) and Delta (B.1.617.2) consist of prefusion S ectodomain residues 1 to 1138 with proline substitutions at amino acid positions 986 and 987, a GGGG substitution at the furin cleavage site (amino acids 682 to 685), and an N-terminal T4 trimerization domain followed by a Strep-tag II (32). Spike was expressed in HEK 293 FreeStyle cells and purified using Strep-TactinXT Superflow high-capacity 50% suspension according to the manufacturer’s protocol by gravity flow (IBA Life Sciences).

N protein was obtained from the James lab at the MRC Laboratory of Molecular Biology (LMB), Cambridge, UK. The N protein is a truncated construct of the SARS-CoV-2 N protein comprising residues 48 to 365 with an N-terminal uncleavable hexahistidine tag. N was expressed in Escherichia coli using autoinducing medium for 7 h at 37°C and was purified using immobilized metal affinity chromatography (IMAC), size exclusion, and heparin chromatography.

### Spike IgG titers by ELISA.

ELISA was carried out as previously described (17). All sera were heat-inactivated at 56°C for 30 min before use in the in-house ELISA. High-binding ELISA plates (Corning; 3690) were coated with antigen (N or spike [WT or Delta]) at 3 μg/mL (25 μL per well) in phosphate-buffered saline (PBS) overnight at 4°C. Wells were washed with PBS-T (PBS with 0.05% Tween 20) and then blocked with 100 μL 5% milk in PBS-T for 1 h at room temperature. Wells were emptied, and a titration of serum starting at 1:50 and using a 6-fold dilution series in milk was added and incubated for 2 h at room temperature. Control reagents included CR3009 (2 μg/mL), CR3022 (0.2 μg/mL), negative-control plasma (1:25 dilution), positive-control plasma (1:50), and blank wells. Wells were washed with PBS-T. Secondary antibody was added and incubated for 1 h at room temperature. IgM was detected using goat anti-human IgM-HRP (horseradish peroxidase) (1:1,000) (Sigma; A6907), and IgG was detected using goat anti-human Fc-AP (alkaline phosphatase) (1:1,000) (Jackson; 109-055-098). Wells were washed with PBS-T, and either AP substrate (Sigma) was added and read at 405 nm (AP) or 1-step TMB (3,3′,5,5′-tetramethylbenzidine) substrate (Thermo Scientific) was added and quenched with 0.5 M H_2_S0_4_ before reading at 450 nm (HRP). Half-maximal binding (EC_50_) was calculated using GraphPad Prism. Measurements were carried out in duplicate.

### SARS-CoV-2 pseudotyped virus particle preparation.

Pseudotyped HIV-1 virus incorporating the SARS-CoV-2 spike protein (either wild-type, B.1.1.7, B.1.351, B.1.621, B.1.617.2, or B.1.1.529) was prepared as previously described (18). Viral particles were produced in a 10-cm dish seeded the day prior with 5 × 10^6^ HEK 293T/17 cells in 10 mL of complete Dulbecco’s modified Eagle’s medium (DMEM-C; 10% fetal bovine serum [FBS] and 1% Pen/Strep) containing 10% (vol/vol) FBS, 100 IU/mL penicillin, and 100 μg/mL streptomycin. Cells were transfected using 90 μg of PEI-Max (1 mg/mL; Polysciences) with 15 μg HIV-luciferase plasmid, 10 μg HIV 8.91 gag/pol plasmid, and 5 μg SARS-CoV-2 spike protein plasmid (33, 34). The supernatant was harvested 72 h posttransfection. Pseudotyped virus particles were filtered through a 0.45-μm filter and stored at −80°C until required.

### Neutralization assay with SARS-CoV-2 pseudotyped virus.

Serial dilutions of serum samples (heat-inactivated at 56°C for 30 min) were prepared with DMEM (25 μL) (10% FBS and 1% Pen/Strep) and incubated with pseudotyped virus (25 μL) for 1 h at 37°C in half-area 96-well plates. Next, HeLa cells stably expressing the ACE2 receptor were added (10,000 cells/25 μL per well), and the plates were left for 72 h. Infection levels were assessed in lysed cells with the Bright-Glo luciferase kit (Promega), using a Victor X3 multilabel reader (Perkin Elmer). Each serum sample was run in duplicate and was measured against the five SARS-CoV-2 variants within the same experiment using the same dilution series.

### Statistical analysis.

Analyses (Spearman rank correlation and Wilcoxon matched-pair signed rank test) were performed using GraphPad Prism v.8.3.1.
