# Supplementary material for: Broad Neutralization of SARS-CoV-2 Variants, Including Omicron, following Breakthrough Infection with Delta in COVID-19-Vaccinated Individuals
Source: mBio. 2022 Mar 17;13(2):e03798-21. doi: 10.1128/mbio.03798-21 (PMC9040729; doi:10.1128/mbio.03798-21)
Supplement: TABLE S1 [file mbio.03798-21-st001.pdf]

Patient number	Vaccine type	Days post onset of symptoms	Days since last vaccine	Severity of disease	Age	Co-morbidities	Any immunosuppression/immune dysfunction	S IgG EC50 WT	S IgG EC50 Delta	S IgM EC50 WT	S IgM EC50 Delta	N IgG OD	N IgG fold change above background	WT ID50	B.1.1.7 ID50	B.1.351 ID50	B.1.621 ID50	B.1.617.2 ID50	WT ID50	B.1.617.2 ID50	B.1.1.529 ID50
														Data in figure 2					Data in figure 3		
9	AZ	18	69	1	42	Hypothyroid	Nil	1386	1318	117	104	1.532	15.6	1782	3784	415	1274	1019	1117	875	146
10	AZ	1	118	4	82	CCF, CKD4, T2DM, CVA	Nil	<25	25	25	<25	0.357	3.6	28	<25	<25	26	<25			
10	AZ	9	118	4	82	CCF, CKD4, T2DM, CVA	Nil	24665	23955	37	26	1.081	11	15996	27416	24972	23823	20277			
10	AZ	15	118	4	82	CCF, CKD4, T2DM, CVA	Nil	14671	15440	25	25	1.125	11.4	8472	13521	10348	6546	14256	7638	18967	5012
11	AZ	7	100	4	83	Gastric GIST+mets, COPD	imatinib	<25	<25	<25	<25	0.257	2.6	<25	<25	<25	<25	<25			
11	AZ	15	100	4	83	Gastric GIST+mets, COPD	imatinib	1861	2180	74	57	1.469	14.9	794	4325	1754	2477	658	1122	977	46
12	AZ	13	94	0	66	Asthma, OSA	nil	10022	10815	714	717	1.046	10.6	14997	8395	12472	13868	7551	8054	13305	7980
13	AZ	12	68	0	89	metastatic gastric cancer, bladder cancer, frail	Nill (palliative)	<25	<25	25	<25	0.304	3.1	32	<25	<25	<25	26			
14	AZ	13	102	0	61	T2DM	Nil	17905	18601	696	352	1.109	11.3	48753	17701	4966	15885	15812	6266	19055	17947
15	AZ	14	106	0	74	?	Nil	26506	30040	548	275	0.617	6.3	6353	17498	10093	7278	8770			
16	AZ	21	74	0	68	Nil	Nil	10902	11263	59	45	1.543	15.7	6310	10520	5358	6577	13305			
17	AZ	15	57	1	88	CKD, T2DM, Asthma	Nil	6245	6615	774	568	1.315	13.4	1837	8590	5998	7362	6237			
17	AZ	20	57	1	88	CKD, T2DM, Asthma	Nil	5713	6471	1023	782	1.51	15.3	2113	8933	5781	6934	4102			
18	AZ	7	109	4	63	HTN, T2DM	Nil	20616	21496	372	255	0.145	1.5	8091	7980	8967	7889	6223			
18	AZ	18	109	4	63	HTN, T2DM	Nil	11651	14268	543	409	1.722	17.5	54954	17100	3741	24099	24547			
19	AZ	1	167	1	103	Frail	Nil	<25	<25	<25	<25	0.176	1.8	<25	<25	<25	<25	<25			
19	AZ	11	167	1	103	Frail	Nil	102	249	258	327	1.495	15.2	500	861	<25	65	1710			
20	AZ	8	71	0	71	T2DM, dialysis, HTN, cured HCV	Nil	2368	2967	61	62	0.781	7.9	5572	1690	1132	1300	1109			
20	AZ	35	72	0	71	T2DM, dialysis, HTN, cured HCV	Nil	2548	3111	87	76	1.391	14.1	4875	4550	3334	2851	995			
21	AZ	4	72	1	56	hepatobilary cancer	Nil	19427	19882	306	199	0.943	9.6	9616	7962	5848	7499	7834			
21	AZ	9	72	1	56	hepatobilary cancer	Nil	21359	21777	450	481	1.342	13.6	16634	22803	16904	21086	25410			
22	AZ	3	109	4	33	Epilepsy	Nil	<25	<25	<25	<25	0.254	2.6	25	25	25	25	<25			
22	AZ	9	109	4	33	Epilepsy	Nil	14149	14481	604	521	0.661	6.7	2661	11455	12942	11588	7161			
22	AZ	19	109	4	33	Epilepsy	Nil	19657	18592	381	360	1.492	15.1	2979	3606	5819	5085	3412	2825	9506	6516
23	AZ	2	108	0	96	Alzheimer's, HTN, CVA	Nil	93	93	43	26	0.143	1.5	<25	102	131	132	89			
23	AZ	14	167	0	96	Alzheimer's, HTN, CVA	Nil	48122	46450	6820	6882	0.873	8.9	31189	35892	7161	41210	36308	22336	51880	22336
25	AZ	21	33	0	25	None	Nil	3642	4025	301	250	0.534	5.4	2710	1374	4420	2377	2773			
26	AZ	43	77	1	67	Rheumatoid arthritis	methotrexate, Certolizumab	948	924	751	831	0.792	8	2427	1972	2460	1828	2355			
26	AZ	53	77	1	67	Rheumatoid arthritis	methotrexate, Certolizumab	1320	1662	323	787	0.688	7	2228	1416	3475	3540	10691			
27	AZ	37	165	4	26	SMC1A mutation, epilepsy, developmental delay	Nil	1100	1072	25	25	0.937	9.5	1811	1910	1542	1361	1714			
28	AZ	5	41	4	59	t2DM	Nil	3397	3784	109	117	0.912	9.3	968	2094	3631	2917	1923			
28	AZ	14	41	4	59	t2DM	Nil	8394	8931	303	436	0.958	9.7	2985	7780	10139	6982	3365	6138	5272	2317
29	AZ	12	125	1	78	T2DM	Nil	27818	28802	182	119	0.9	9.1	14997	6592	8035	11967	25410			
39	AZ	8	29	1	20	Nil	Nil	259	328	25	25	0.77	7.8	2328	2443	1663	2477	2667			
41	AZ	89	40	0	38	Crohn's	Nil	28	43	25	<25	0.465	4.7	186	228	307	274	822			
42	AZ	-1	120	0	75	HTN, Bladder outflow obstruction, Anticoagulated	Nil	1434	1240	25	25	0.915	9.3	360	2477	1611	2028	1799			
1	Pfizer	18	58	4	66	Lupus	Aza, HCQ, Pred	2127	2322	67	37	1.517	15.4	4159	6934	698	2399	7834	3963	11858	146
1	Pfizer	25	58	4	66	Lupus	Aza, HCQ, Pred	562	1102	620	711	1.335	13.6	1671	689	508	577	1758			
2	Pfizer	12	146	3	83	AF, HTN, Chol	Nil	6042	6452	607	498	0.478	4.9	1884	3467	3917	2014	3428	2104	5000	3041
2	Pfizer	16	146	3	83	AF, HTN, Chol	Nil	8020	7847	1382	1103	1.101	11.2	4529	11695	12050	7745	10162			
3	Pfizer	15	76	0	49	HTN, T2DM, dialysis	nil	5712	6885	703	660	0.638	6.5	2624	7998	5729	5821	15849	3589	10000	2588
4	Pfizer	1	89	0	63	Liver cirrhosis	Nil	115	146	25	25	0.231	2.3	<25	95	156	87	<25			
4	Pfizer	6	89	0	63	Liver cirrhosis	Nil	74	64	25	25	0.249	2.5	27	61	84	80	<25			
4	Pfizer	16	89	0	63	Liver cirrhosis	Nil	6525	6827	334	205	0.802	8.1	1718	7870	6067	7379	5943	2000	9099	3890
5	Pfizer	6	127	0	95	COPD, asthma, BPH	Nil	25	25	<25	<25	0.188	1.9	<25	<25	<25	32	<25			
5	Pfizer	11	127	0	95	COPD, asthma, BPH	Nil	2744	2764	43	34	0.601	6.1	1919	3192	2685	2924	1766			
6	Pfizer	13	100	4	57	Renal transplant	Tacrolimus, prednisolone, mycophenolate	<25	<25	48	56	0.398	4	<25	<25	<25	<25	<25			
6	Pfizer	16	100	4	57	Renal transplant	Tacrolimus, prednisolone, mycophenolate	25	66	3185	3932	1.168	11.9	7079	3981	3162	5408	47863	3581	10495	210
7	Pfizer	1	134	0	86	HTN	Nil	14221	14012	47	11	1.699	17.2	6622	11749	16866	11272	11246			
8	Pfizer	11	137	1	88	T2DM, Hypertension	Nil	4560	4451	25	25	0.406	4.1	2328	4699	4446	3733	3381			
24	Pfizer	21	105	0	24	Dialysis	Tacrolimux, MMF, Pred	10911	11430	1106	1851	1.353	13.7	5224	7925	15942	8892	25293	3357	24660	7674
30	Pfizer	0	171	4	75	OSA, polychondritis	Methotrexate	229	234	56	48	0.352	3.6	492	327	196	319	279			
30	Pfizer	8	171	4	75	OSA, polychondritis	Methotrexate	4347	4666	1313	1232	1.551	15.7	7482	6823	973	1265	8128			
31	Pfizer	3	154	0	76	Stroke	Nil	9949	11028	483	387	0.133	1.4	8260	8147	3083	6209	7311			
31	Pfizer	13	154	0	76	Stroke	Nil	48347	46322	708	588	1.121	11.4	80724	100000	84918	53088	81283			
32	Pfizer	16	149	0	79	t2DM, frail	Nil	4099	3966	369	632	0.249	2.5	3289	5598	5943	4966	3707	1262	10593	1959
33	Pfizer	10	173	0	95	T2DM, CKD3, COPD, oesophagitis (chronic)	Nil	30	49	78	61	0.139	1.4	25	<25	25	25	<25			
34	Pfizer	34	132	3	79	Polycystic kidneys, HTN, gout	Nil	7351	7389	904	2758	1.277	13	7962	11272	3811	5272	4797			
35	Pfizer	34	149	4	80	Cardiovascular disease, COPD	Nil	1811	2310	395	2283	1.349	13.7	4808	1486	1476	3273	9057			
36	Pfizer	25	179	1	88	HTN, Angina	Nil	3266	3903	72	64	0.413	4.2	4487	5689	1435	4467	1282			
37	Pfizer	25	119	4	48	Non Hodgkins	Rituximab	<25	<25	<25	<25	0.238	2.4	25	<25	25	47	25			
40	Pfizer	3	116	0	35	Nil	Nil	163	88	26	25	0.237	2.4	267	191	290	377	524			
43	Pfizer	-1	162	0	78	t2DM, Dementia	Nil	12523	15646	169	110	1.567	15.9	11143	34356	27353	19364	12853			
