# Supplementary material for: Broad Neutralization of SARS-CoV-2 Variants, Including Omicron, following Breakthrough Infection with Delta in COVID-19-Vaccinated Individuals
Source: mBio. 2022 Mar 17;13(2):e03798-21. doi: 10.1128/mbio.03798-21 (PMC9040729; doi:10.1128/mbio.03798-21)
Supplement: TABLE S2 [file mbio.03798-21-st002.pdf]

Patient number	Vaccine type	Days post onset of symptoms	Severity of disease	Age	Co-morbidities	S IgG EC50 WT	S IgG EC50 Delta	S IgM EC50 WT	S IgM EC50 Delta	N IgG OD	N IgG fold change above background	WT ID50	B.1.1.7 ID50	B.1.351 ID50	B.1.621 ID50	B.1.617.2 ID50	WT ID50	B.1.617.2 ID50	B.1.1.529 ID50
												Data for figure 2					Data for figure 3		
1	zero	17	5	35	Nil	514	1089	472	677	1.45	14.7	1009	649	475	566	1845	830	2500	90
2	zero	15	5	39	T2DM, Morbid obesity	463	1944	400	1216	1.423	14.4	9908	2153	762	2443	14355	1271	10000	281
3	zero	19	2	82	HTN	312	1218	722	2444	1.737	17.6	1117	1262	1476	1035	8511	916	8730	282
4	zero	13	1 or 2	26	Nil	25	89	1808	3510	1.131	11.5	4325	1607	410	2275	13964			
5	zero	16	5	67	HIV, coarctation	25	48	350	777	1.271	12.9	1644	1300	793	1211	4121	731	7079	356
6	zero	18	1 or 2	28	Nil	397	1226	2746	3461	1.194	12.1	1321	1064	416	1242	6934	1146	7464	231
7	zero	12	1 or 2	37	Nil	25	38	961	1144	0.428	4.3	535	282	301	443	2168			
8	zero	15	4	73	T2DM, HTN, Nephrectomy, MGUS	42	605	1422	1888	1.224	12.4	456	234	1786	1245	2642	262	2259	28
9	zero	15	5	43	Morbidy obesity, T2DM	25	741	1091	1520	1.614	16.4	4645	7211	923	3864	15524	5957	22387	1007
10	zero	13	4	31	Pregnancy	25	25	403	724	0.645	6.5	530	231	211	167	1462	417	1718	52
11	zero	14	1 or 2	34	Nil	<25	<25	25	25	0.238	2.4	<25	<25	36	26	<25			
12	zero	18	1 or 2	55	Nil	<25	<25	<25	<25	0.193	2	<25	<25	<25	<25	<25			
13	zero	14	2	74	OSA	<25	<25	99	155	0.225	2.3	30	<25	<25	26	611	32	681	<25
14	zero	14	1 or 2	27	Asthma	157	557	3139	7575	0.909	9.2	4159	1694	881	3373	21878	1242	15631	283
15	zero	18	5	44	Nil	1070	2622	7337	15574	1.48	15	3327	3013	2213	2979	18793	3999	27669	535
16	zero	13	2	46	Nil	25	25	1282	5627	0.873	8.9	1016	1387	218	773	15996			
17	zero	13	4	25	Nil	25	42	675	913	1.133	11.5	1127	360	137	242	1941	776	2944	46
18	zero	13	4	39	T2DM, pregnant	25	358	1412	2882	0.686	7	1183	3524	526	3062	11967	1189	15959	67
19	zero	22	1 or 2	25	Nil	<25	<25	25	52	0.213	2.2	125	35	<25	41	119	46	215	<25
