# Supplementary figures and images for: Broad Neutralization of SARS-CoV-2 Variants, Including Omicron, following Breakthrough Infection with Delta in COVID-19-Vaccinated Individuals
Source: mBio. 2022 Mar 17;13(2):e03798-21. doi: 10.1128/mbio.03798-21 (PMC9040729; doi:10.1128/mbio.03798-21)

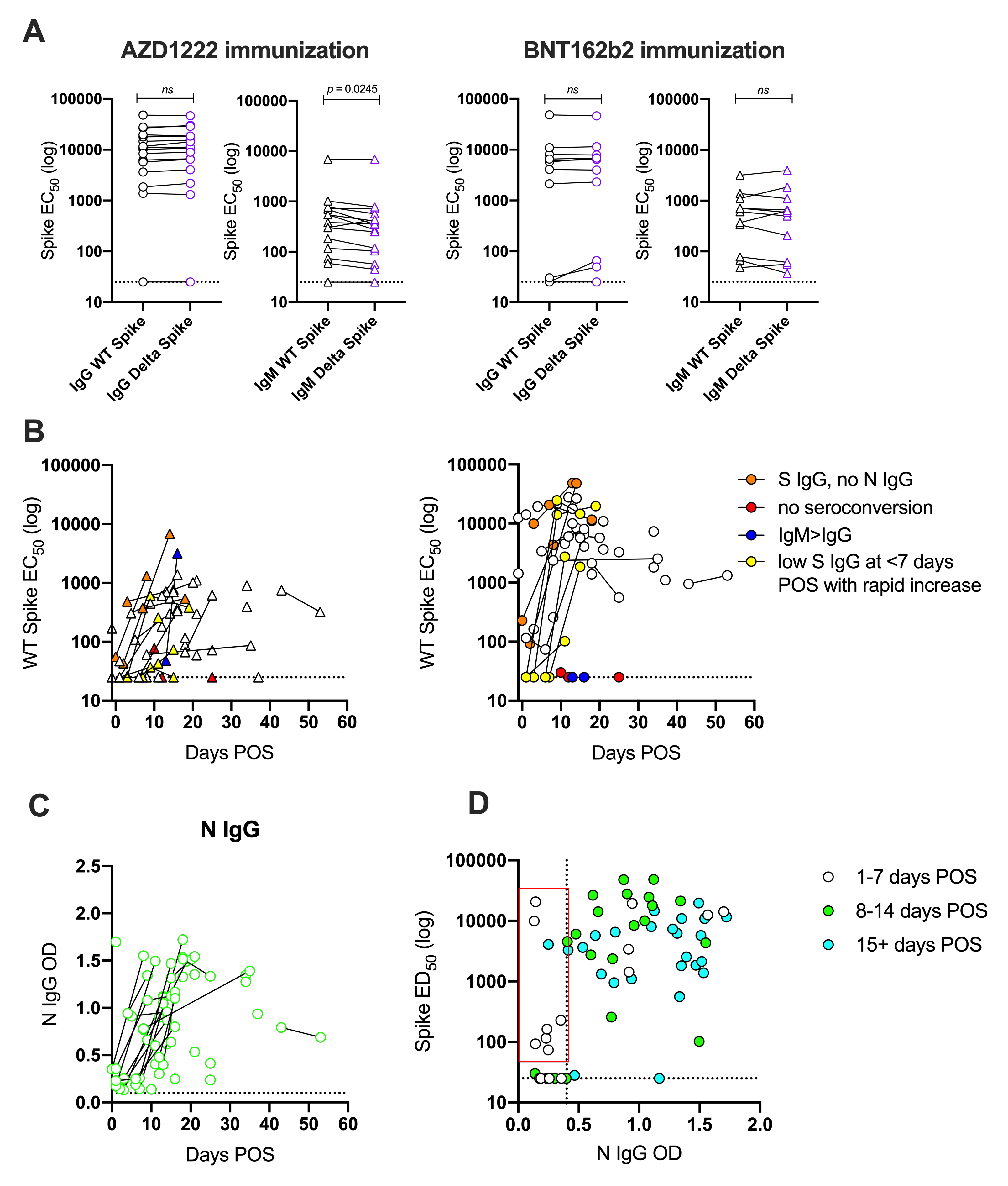

Supplement: FIG S1 [file mbio.03798-21-sf001.tif]

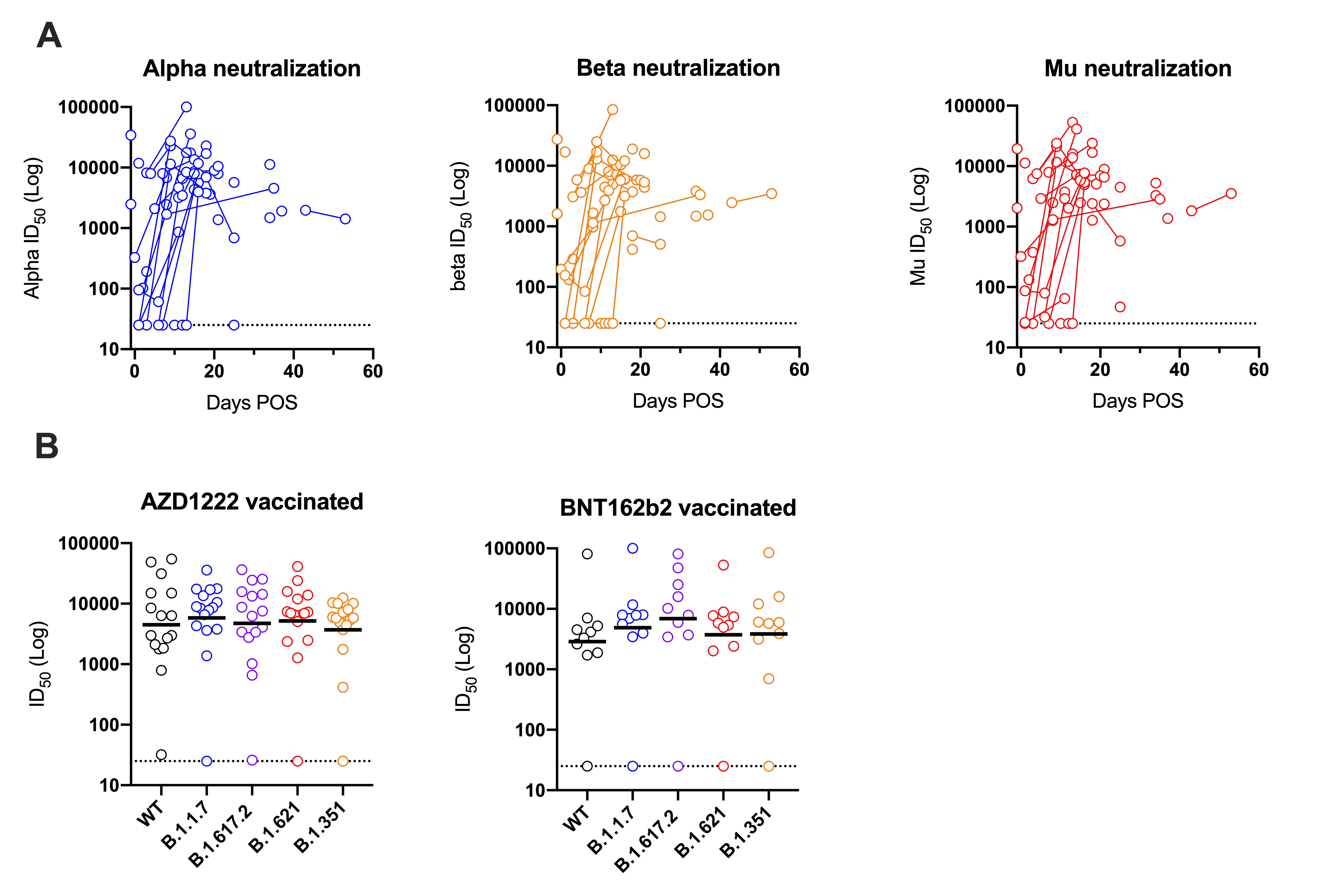

Supplement: FIG S2 [file mbio.03798-21-sf002.tif]
